# The CARE project – study protocol and pilot results from the Polish population

**DOI:** 10.3389/fpsyt.2025.1643722

**Published:** 2025-07-23

**Authors:** Anna Julia Krupa, Silvana Galderisi, Armida Mucci, Tomasz Gondek, Aiste Lengvenyte, Dominika Dudek, Marcin Siwek

**Affiliations:** ^1^ Department of Affective Disorders, Jagiellonian University Medical College, Cracow, Poland; ^2^ University of Campania “Luigi Vanvitelli”, Naples, Italy; ^3^ Institute of Social Studies, University of Lower Silesia, Wroclaw, Poland; ^4^ Department of Emergency Psychiatry and Acute Care, Lapeyronie Hospital, CHU Montpellier, Montpellier, France; ^5^ Institute of Functional Genomics, University of Montpellier, CNRS, INSERM, Montpellier, France; ^6^ Department of Adult Psychiatry, Jagiellonian University Medical College, Cracow, Poland

**Keywords:** schizophrenia, negative symptoms, knowledge, clinical competence, early career psychiatrists, psychiatric trainees

## Abstract

**Introduction:**

Negative symptoms of schizophrenia (SZ) are a critical unmet need of SZ treatment. In the past years, clinical tools were developed and guidance papers for the evaluation and management of negative symptoms of SZ were published. The CARE (Competence and confidence Assessment of early career psychiatrists’ (ECPs) ability to evaluate and manage negative symptoms of SZ) project was designed to examine the competence and confidence of ECPs in assessing and treating negative symptoms of SZ.

**Objective:**

To publish the protocol of the CARE project and a pilot analysis of the data obtained from the Polish sample.

**Methods:**

The CARE project is an international cross-sectional 23-item online survey on competence and confidence in assessing and treating negative symptoms of the ECPs from European countries. This work includes the protocol of the CARE project and a pilot analysis of 140 responses from the Polish ECPs population.

**Results:**

The majority of the participants were trainees (67.2%), not engaged in clinical research (69.3%), reported placement in clinics/wards specialized in SZ care (77.1%) and inclusion of theoretical courses (54.3%) in their specialist training curriculum, and participation in extra-curricular training (62.9%) on the negative symptoms. Few ECPs (6.4%) correctly identified the negative symptoms domains, although the majority of them (55%) reported feeling well-trained to administer and interpret at least one tool for the assessment of the negative symptoms. Respectively, 32.8% and 25.9% reported feeling competent in evaluating and managing the negative symptoms. Specialist status and longer experience were linked to higher likelihood of feeling competent in assessment and management of the negative symptoms. The large majority of ECPs (87.1%) agreed that there should be more emphasis on the negative symptoms of SZ in specialist training. Engagement in clinical research was linked to higher likelihood of correctly identifying the domains of negative symptoms.

**Conclusion:**

The results from the Polish ECPs population indicate a very limited knowledge and preparedness to evaluate and manage negative symptoms of SZ. The CARE study will explore the European ECPs’ gap in knowledge and skills in the evaluation and management of the negative symptoms of SZ to inform future educational actions.

## 1 Introduction

The first psychopathological descriptions of the negative symptoms of schizophrenia (SZ) date back to the 19^th^ century. Both Bleuler and Kraepelin considered the negative symptoms a fundamental part of the SZ syndrome. However, the conceptualization of the negative symptoms was still in development at the time of the introduction of standardized diagnostic classifications such as the Diagnostic and Statistical Manual of Mental Disorders and International Classification of Diseases (1). Positive and first-rank symptoms gained the status of core SZ manifestations, justifying the diagnosis of SZ, while the negative symptoms did not. Nonetheless, several research groups remained dedicated to furthering our understanding of the concept of negative symptoms in SZ, understanding their neurobiological underpinnings, and unraveling their great importance to the functioning and quality of life of people with SZ (1, 2). These efforts resulted in the consensus conference delineating the negative symptom domain of SZ, which includes blunted affect, alogia, asociality, anhedonia, and avolition. According to the consensus statement, the negative symptoms represent a loss of normal functions; they constitute a distinct and often unmet treatment need. The consensus conference also pointed out that the available tools to assess the negative symptoms were no longer in line with the newly established definition of negative symptoms and that new tools applicable in clinical and research setting were needed (2). Indeed, this action was followed by the development and validation of two scales: the Brief Negative Symptoms Scale (BNSS) which was published in 2010 (3), and the Clinical Assessment Interview for Negative Symptoms (CAINS), published in 2013 (4). The BNSS was later translated and validated in many European languages such as Danish, Dutch, French, German, Italian, Norwegian, Polish, Portuguese, Spanish, Turkish (5–15). In 2021, in an effort to provide clinicians with a consistent framework for the diagnosis and management of the negative symptoms of SZ, the Schizophrenia Section of the European Psychiatric Association (EPA) published two guidance papers (16, 17). In 2022, the World Federation of Societies of Biological Psychiatry (WFSBP) and Canadian Network for Mood and Anxiety Treatments (CANMAT) Taskforce created guidelines for the clinical use of nutraceuticals and phytoceuticals for psychiatric disorders, which included specific guidance for the management of the negative symptoms of schizophrenia (18). However, it remains unclear whether these actions have influenced the specialist training in psychiatry and/or the routine clinical practice. Hence, the CARE (Competence and confidence Assessment of early career psychiatrists’ ability to evaluate and manage negative symptoms of schizophrenia) project was designed to explore the competence and confidence of early career psychiatrists (ECPs) in assessing and treating the negative symptoms of SZ.

The goal of the present work is to present the protocol of the CARE project and the pilot analysis of the Polish ECPs’ survey on competence and confidence in evaluating and managing SZ negative symptoms.

## 2 Methods

### 2.1 Hypotheses and aims of the project

The CARE project is a short-term study spanning between September 2024 to September 2025. The general hypotheses for the CARE project are:

1. European ECPs do not receive sufficient training on negative symptoms of SZ.
2. The insufficient training results in poor levels of knowledge and sense of competence as well as a need for more emphasis on negative symptoms of SZ.

To verify these hypotheses the CARE project was designed to obtain data on characteristics of the received specialist training, self-reported sense of competence/attitude toward consulting specific patients groups, knowledge/skills with regard to negative symptoms and the level of implementation of existing guidance among European ECPs. The main goal is to provide a general European perspective on the above issues, hence the project does not set out to obtain a specific number of individual responses, but a sufficient number of independent observations from as much as possible European region countries.

### 2.2 Study design

The CARE project is an international cross-sectional online ECPs’ survey, performed with the support of the EPA Early Career Psychiatrists Committee (ECPC). This survey was developed in cooperation between researchers from the Jagiellonian University Medical College, the EPA ECPC, and the EPA Schizophrenia Section members who had contributed to the development of the EPA Guidance papers on the assessment and treatment of negative symptoms (16, 17), as well as to the introduction and validation in European Countries of the Brief Negative Symptom Scale (BNSS) (19), one of the two scales developed by members of the Consensus conference (2).

### 2.3 Survey construction

The questions included in the survey were based on EPA and WFSBP guidance papers (16–18). The survey consisted of 23 questions: a) 14 single-answer questions (5 on demographic data, 3 on the received training, 4 verifying ECPs’ knowledge on the negative symptoms, 2 assessing ECPs’ familiarity with the EPA and WFSBP guidance papers); b) 7 five-item Likert scales (6 for evaluating the sense of competence and the attitude toward management of patients with negative symptoms of schizophrenia, as well as patients with various mental disorders; 1 to assess how often ECPs use clinical tools to assess negative symptoms of schizophrenia); c) 2 multiple-choice questions to assess ECPs’ knowledge on SZ negative symptoms and their ability to apply specific clinical tools to measure them. Following the survey completion, an educational component was made available to show the correct answers to the knowledge-related questions, and provide references for the EPA and WFSBP publications (**Supplementary Data 1**). The variables included in the survey are presented in **Table 1**.

**Table 1:** Variables included in the CARE study survey.

Demographic	Characteristics of the received training	Declared sense of competence/attitude toward consulting specific patients groups	Knowledge/skills	Implementation
Sex	Theoretical courses on the negative symptoms in the curriculum of specialist training	Evaluating negative symptoms	Identification of specific negative symptoms domains	Use of tools to evaluate negative symptoms in clinical practice
Professional status (trainee/specialist with less than 5 years of clinical practice after specialty/specialist under 40 years of age	Placements in specialized schizophrenia clinics/wards in the curriculum of specialist training	Managing negative symptoms	Preparedness to administer and interpret clinical tools to assess negative symptoms	Familiarity with EPA guidance on assessment and treatment of negative symptoms
Country in which the ECP works	Extracurricular theoretical or practical training in the negative symptoms of schizophrenia	Consulting people with negative vs. positive symptoms of schizophrenia	Familiarity with EPA guidance with regard to pharmacological interventions	Familiarity with the WFSBP/CANMAT guidelines on nutraceutical and phytoceutical interventions
Years of experience in mental health care system		Consulting people with major depressive disorder, schizophrenia, bipolar disorder, Personality disorder, Schizophrenia with persistent negative symptoms Substance dependence	Familiarity with EPA guidance with regard to non-pharmacological interventions	Expression of the need for more emphasis and/or time on the evaluation and management of the negative symptoms in the specialist training
Engagement in clinical research			Familiarity with WFSBP/CANMAT guidelines on nutraceutical and phytoceutical interventions	

### 2.4 Study group

The eligibility criteria were: 1) being an ECP, according to the following definition: A trainee in psychiatry, a psychiatrist under 40 years of age, or a psychiatrist less than 5 years after specialty; 2) working in one of the World Health Organization (WHO) European region countries (20).All respondents confirmed their awareness of the fact that the survey could be completed only once.

### 2.5 Data collection

The survey was constructed and distributed in English via the Google Forms tool. The survey is accessible via the invitation link (https://forms.gle/31Fxum7vB4osJ8wM7) or the related QR code. It was initially (from December 2024 to February 2025) disseminated among Polish ECPs, who were asked to provide feedback on whether the questions were clear and comprehensible. A pilot analysis of their responses was performed to verify the initial results and ensure understandability of the survey. None of the respondents complained about the level of clarity of this survey. The international dissemination of the survey is ongoing (from February to June 2025), via collaboration between its creators and national coordinators affiliated with the EPA, National Psychiatric Associations (NPA), or European Federation of Psychiatric Trainees (EFPT) from various European countries. The ECPs are reached through: 1) the websites, mailing lists, and social media of the EPA, NPAs of specific European countries, as well as the EFPT; 2) other online platforms dedicated to psychiatrists and 3) via announcements at the briefings and staff meetings at mental health facilities and at conferences for psychiatrists. Participants are encouraged to disseminate the questionnaire among their colleagues who fulfilled the inclusion criteria. The results obtained from all ECPs will be analyzed when the target of at least 25 independent observations from participants working in the most populated (>15 million; Russia, Germany, United Kingdom, France, Italy, Spain, Ukraine, Poland, Romania, Netherlands) European countries, and 15 independent observations from participants from less populated countries (>1<15 millions; Belgium, Sweden, Czech Republic, Portugal, Greece, Hungary, Austria, Belarus, Switzerland, Bulgaria, Serbia, Denmark, Finland, Norway, Slovakia, Ireland, Croatia, Bosnia and Herzegovina, Moldova, Lithuania, Albania, Slovenia, Latvia, North Macedonia, Estonia) will be reached. Not all WHO European region countries could be included in the study, due to restrictions enforced by the funding agency (i.e., Russian Federation inclusion was not allowed) or for the present war conditions (i.e., Ukraine). Therefore, the aim is to obtain the planned number of responses from at least 7 of the most populated and at least 10 of the less populated European countries. The responses from the countries which will not reach the minimum number of respondents will not be included in the analysis.

### 2.6 Ethics approval and storage

This study was approved by the Research Ethics Committee of the Jagiellonian University Medical College (Krakow, Poland). Participants were informed of the aims of the study, that the survey was completely anonymous, and their participation voluntary; that if they changed their mind, they could interrupt filling in the survey at any point, and that only complete responses would be collected.

To comply with the General Data Protection Regulation, only anonymous data were collected. Once the collection process is completed, the data will be stored with alignment with appropriate and technical and organizational measures in place to safeguard them. All data collected are processed in compliance with the data protection regulations of the European Union and the Republic of Poland.

### 2.7 Statistical analysis

#### 2.7.1 Protocol of the analyses for the CARE study

IBM SPSS software was used for statistical analysis. Categorical variables were analyzed using frequency distribution, while continuous variables using means/medians. Contingency tables and *X* ^2^ tests (categorical variables) and analysis of variance (ANOVA)(for continuous variables) will be used to investigate the associations between demographic and training characteristics and main study outcomes (declared sense of competence/attitude toward consulting specific patients groups, knowledge and implementation) (**Table 1**). Next, logistic and linear regression analyses will be performed to verify whether demographic and training characteristics are predictive of the main study outcomes (which will constitute the dependent variables for these analyses). The significance level for these analyses will be defined as a two-tailed p-value < 0.05.

#### 2.7.2 Analyses of the pilot results from Polish population

The main outcomes were the level of competence and sense of confidence in evaluating and managing the negative symptoms of SZ declared by the Polish ECPs and whether they agreed that the specialist training should put more emphasis and/or time on the evaluation and management of the negative symptoms of SZ. The Kolmogorov–Smirnov test was use to assess the data distribution. The Mann–Whitney tests (quantitative variables) or *X* ^2^ tests (qualitative variables) with Bonferroni correction for multiple comparisons, if needed, were performed to verify potential links between demographic variables and training/work experience and the knowledge, the self-reported sense of competence in evaluating and managing negative symptoms of SZ as well as the feeling of being well-trained to administer a tool for the assessment of the negative symptoms of schizophrenia. Odds ratio (OR) were reported for the analyses yielding significant results for 2x2 contingency tables and for these parts comparisons of 3x2 contingency tables, which differed significantly. For this pilot analysis the Likert scales responses were grouped as positive (strongly agree/agree), neutral (neither agrees nor disagrees) or negative (strongly disagrees/disagrees).

## 3 Results of the pilot analysis

### 3.1 General group characteristics

**Figure 1:**
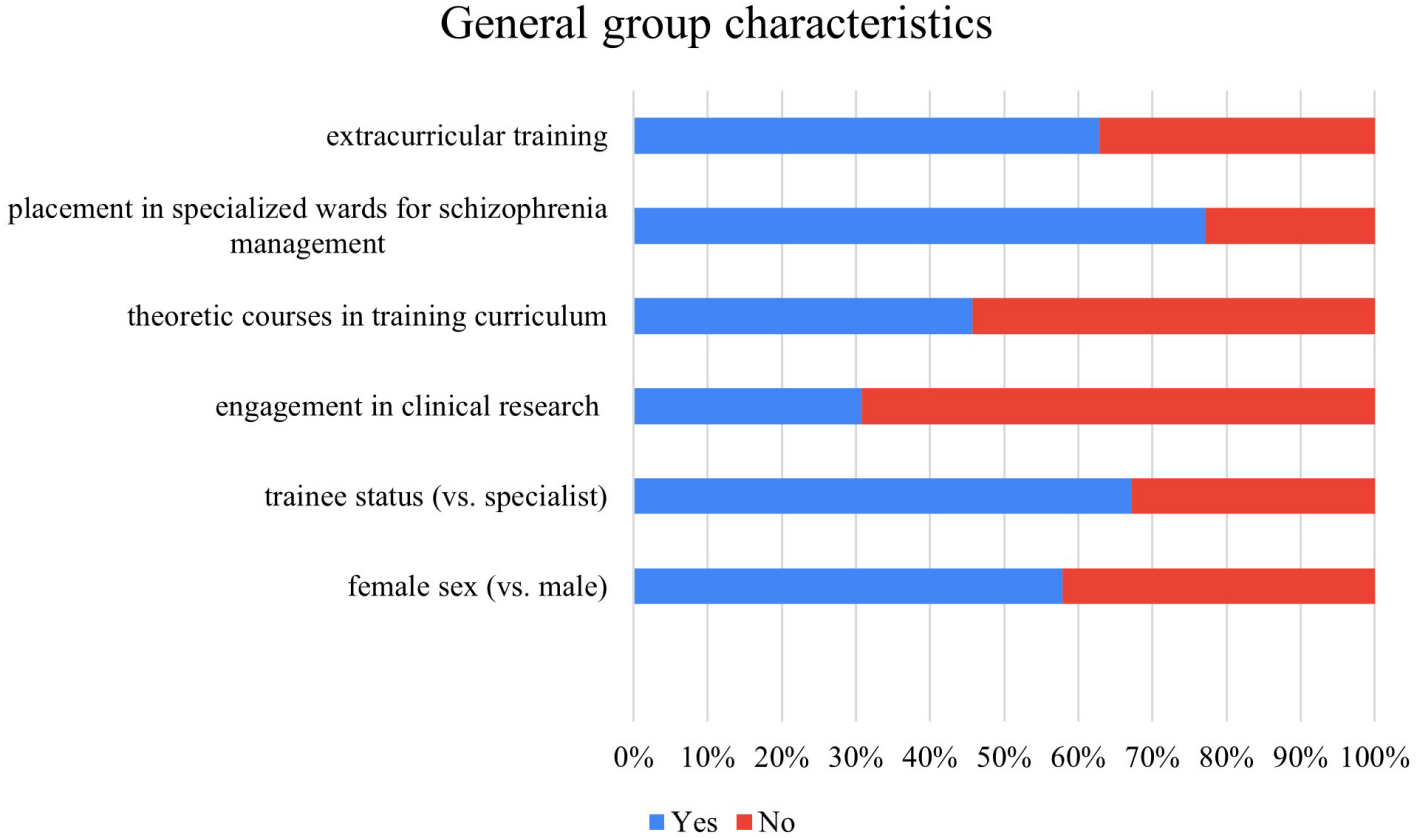
General group characteristics.

This pilot analysis is based on the data from 140 ECPs from Poland. Group characteristics are presented in **Table 2** and illustrated in **Figure 1**. Slightly over a half of ECPs were females (59.7%). The majority of the participants were trainees (67.2%). The median number of years of experience in the mental health care system was 4 (interquartile range 2-7). Less than one third of the respondents reported being engaged in clinical research (30.7%). Over half (54.3%) of ECPs reported that their specialist training included theoretical courses on negative symptoms of SZ. Most participants (77.1%) reported that they had placements in clinics or wards specializing in SZ care. Additionally, 62.9% had attended extracurricular training on the negative symptoms of SZ.

**Table 2:** General group description.

Demographic and training/work experience characteristics	Number of participants (% of the whole group)
Sex (Female/Male) (%)	81 (57.9%)/59 (42.1%)
Number of years of experience in the mental health system [median (IQR)]	4 (2-7)
Trainee/specialist in adult or child and adolescent psychiatry (%)	94 (67.2%)/46 (32.8%)
Engagement in clinical research yes/no (%)	43 (30.7%)/97 (69.3%)
Theoretical courses on the negative symptoms of schizophrenia included in the specialist training curriculum yes/no (%)	64 (54.3%)/76 (45.7%)
Placements in clinics/wards specialized in schizophrenia care and management included in the specialist training curriculum yes/no (%)	108 (77.1%)/32 (22.9%)
Participated in additional theoretical or practical training in the negative symptoms of schizophrenia assessment and management (outside of specialist training programme) yes/no (%)	88 (62.9%)/52 (37.1%)

IQR, Interquartile range.

### 3.2 ECPs knowledge and sense of competence in evaluation and management of the negative symptoms of SZ

**Figure 2:**
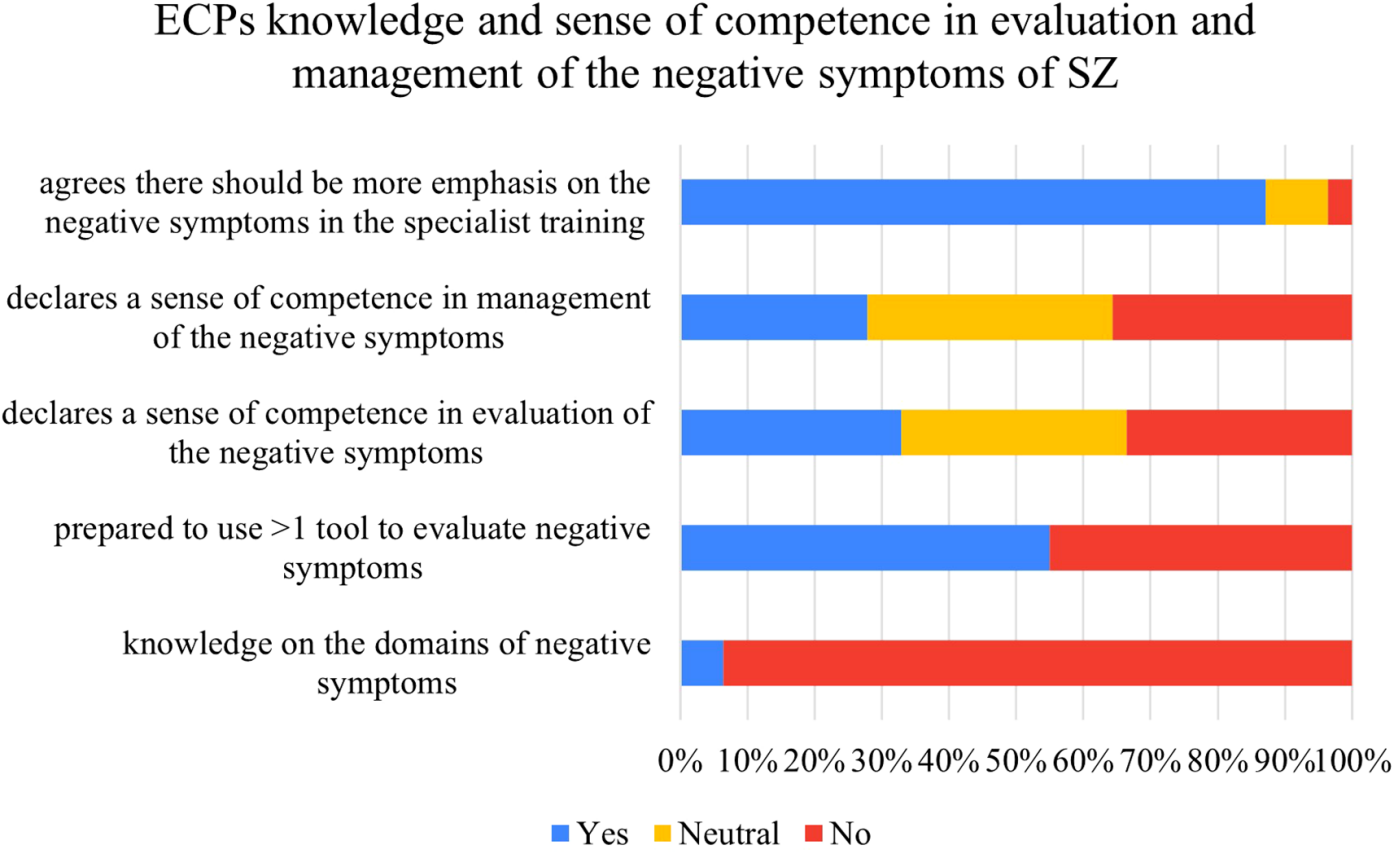
ECPs knowledge and sense of competence in evaluation and management of the negative symptoms of SZ.

The levels of knowledge and confidence in evaluating and managing the negative symptoms of SZ are presented in **Table 3** and **Figure 2**. Only 6.4% of Polish ECPs correctly identified the domains of negative symptoms. Over a half of respondents reported feeling well-trained to administer and interpret at least one tool assessing the negative symptoms of SZ (55%). Only 32.8% and 25.9% agreed that they felt competent in evaluating or managing the negative symptoms of SZ, respectively. The large majority of respondents (87.1%) agreed that there should be more emphasis on the negative symptoms of SZ during specialist training.

**Table 3:** Knowledge on the domains of negative symptoms of SZ, declared levels of sense of competence in evaluation and management of the negative symptoms of SZ, agreement that there should be more emphasis on the negative symptoms of SZ in specialist training and knowledge of tools to assess the negative symptoms of schizophrenia.

Variables	Number of participants (% of the whole group)
Correct choice of the responses to the question on the domains of the negative symptoms of SZ yes/no (%)	9 (6.4%)/131 (93.6%)
Report of feeling well-trained to administer and interpret at least one tool for the assessment of the negative symptoms of SZ yes/no (%)	77 (55%)/63 (45%)
Report of a sense of competence in evaluation of the negative symptoms of SZ agrees/neither agrees nor disagrees/disagrees (%)	46 (32.8%)/47 (33.6%)/47 (33.6%)
Report of a sense of competence in management of the negative symptoms of SZ agrees/neither agrees nor disagrees/disagrees (%)	39 (25.9%)/51 (36.4%)/50 (37.7%)
Agreement that there should be more emphasis on the negative symptoms of SZ in specialist training agrees/neither agrees nor disagrees/disagrees (%)	122 (87.1%)/13 (9.3%)/5 (3.6%)

### 3.3 Relationships between ECPs’ individual characteristics and training and levels of knowledge, and sense of competence in evaluating and managing the negative symptoms of SZ

Associations of ECPs’ individual characteristics and training with the levels of knowledge and sense of competence in evaluating and managing the SZ negative symptoms are displayed in **Table 4**. Engagement in clinical research was the only demographic/training-related variable linked to significantly higher likelihood of correctly identifying the negative symptom domains of SZ (**Table 4**). Inclusion of theoretical courses on negative symptoms of SZ in specialist training was linked to higher likelihood of feeling well-trained to use at least one tool for the evaluation of these symptoms (**Table 4**). Longer experience in the mental health care and specialist status were associated with higher declared sense of competence in evaluation and management of negative symptoms of SZ (**Table 4**). Participation in extra-curricular training was linked to higher likelihood of feeling competent in management of negative symptoms (**Table 4**). Finally, engagement in clinical research was linked to lower likelihood of considering necessary more training on the negative symptoms of SZ in the curriculum (**Table 4**).

**Table 4:** General characteristics linked to the levels of knowledge, sense of competence in evaluation and management as well as reported need for more emphasis in the training on the negative symptoms.

	Correct choice of the responses to the question identifying the domains of negative symptoms of SZ	Report of feeling well-trained to use at least one tool for the assessment of the negative symptoms of SZ	Sense of competence in evaluation on the negative symptoms of SZ	Sense of competence in management on the negative symptoms of SZ	Agreement on need for more training on the negative symptoms of SZ in the curriculum
Sex	p = 0.734 ^a^	p = 0.594 ^a^	p = 0.113 ^a^	p = 0.065 ^a^	p > 0.99 ^a^
Years of experience in the mental health system	p = 0.765 ^b^	p = 0.695 ^c^	p = 0.007 ^c^	p < 0.001^c^	p = 0.907 ^c^
Trainee or specialist status	p = 0.975 ^a^	p = 0.329 ^a^	p = 0.063 ^a^ p = 0.023 ^d^ OR = 2.75 (95% CI 1.13-6.7)	p = 0.006 ^a^ p = 0.004 ^d^ OR = 4.06 (95% CI 1.51-10.87)	p = 0.061 ^a^
Engagement in clinical research	p = 0.024 ^a^ OR = 5.08 (95% CI 1.21-21.39)	p = 0.217 ^a^	p = 0.217 ^a^	p = 0.055 ^a^	p = 0.005 ^a^ p = 0.003 ^e^ OR = 0.19 (95% CI 0.05-0.55)
Theoretical courses on the negative symptoms of schizophrenia	p = 0.509 ^a^	p = 0.003 OR = 2.87 (95% CI 1.43-5.75)	p = 0.435 ^a^	p = 0.228 ^a^	p = 0.066 ^a^
Placements in clinics/wards specialized in schizophrenia care and management	p = 0.684 ^a^	p = 0.145 ^a^	p = 0.865 ^a^	p = 0.918 ^a^	p = 0.591 ^a^
Additional theoretical or practical training in the negative symptoms of schizophrenia assessment and management	p = 0.727 ^a^	p = 0.833 ^a^	p = 0.063 ^a^	p = 0.015 ^a^ p = 0.006 ^d^ OR 3.61 (95% CI 1.43-9.14)	p > 0.99 ^a^

p-values are presented in the table for all comparisons, for comparisons indicating significant associations the size effect as measured by odds ratio was reported.

a *X*2- test.

b Mann-Whitney U test.

c Kruskal-Wallis test.

d significant difference in comparison between subjects responding positively vs. negatively.

e significant difference in comparison between subjects responding neutrally vs. negatively 95% CI – 95% confidence interval, OR = odds ratio.

## 4 Discussion

The obtained preliminary results indicate that the vast majority of Polish ECPs (over 90%) lack an updated knowledge of SZ negative symptoms domains as defined in the 2006 expert consensus and EPA guidelines (2, 16). This finding aligns with the literature suggesting that basic factual knowledge is often undervalued in medical training (21). Larger scale studies, conducted in the United States assessing the effects of psychiatric residency programs, showed that the level of trainees’ knowledge increased over the years of residency but only for some of the sub-competencies (psychopathology, psychotherapy, somatic therapies but not development, clinical neuroscience, or practice of psychiatry) (22). In contrast, our preliminary results did not show any link of the length of experience in mental health care and of trainee/specialist status with actual knowledge about negative symptom domains. Interestingly, ECPs’ engagement in clinical research was the only factor significantly associated with accurate knowledge of negative symptom domains. It’s worth noting that less than one third of Polish ECPs confirmed engagement in clinical research. This might be due to the fact that in Poland the majority of psychiatric research is conducted in the major academic centers (23), while specialist training is available not only in university hospitals but also in peripheral placements outside the academic context accredited by the Centre of Postgraduate Medical Education (24).

Moreover, it has been reported that the psychiatric training programmes in psychopathology often lack an appropriate evaluation of how knowledge translates into real-world setting behaviors and outcomes (25). Indeed, our analysis shows that only 55% of ECPs feels well-trained to use at least one tool for the assessment of the negative symptoms of SZ, despite the fact that skills in application and interpretation of standardized clinical tools is listed as a part of the specialist training in psychiatry in Poland (26).

Of note, less than one third of the ECPs reported a sense of competence in evaluating the negative symptoms of SZ. Notably, the sense of competence was associated with the length of clinical experience but not the actual knowledge. About one fourth of ECPs reported a sense of competence in the management of the negative symptoms of SZ. This result seems to be in agreement with the data indicating overall low levels of sense of competence in healthcare professionals in the management of psychotic patients (27).

The striking difference between the levels of knowledge (6.4%) and ECPs’ self-reported sense of competence in evaluating (32.8%) and managing (25.9%) mirrors findings from other domains, such as eating disorders for mental health professionals (28) or psychosis for general practitioners (29). In line with these data, weak correlations between self-confidence and knowledge test results have been reported for physicians of other specialties (30). There is an even larger discrepancy between the levels of knowledge (6.4%) and declared preparedness to administer and interpret at least one tool to evaluate NS (55%). This could be due to the fact that 1) most old and widely known rating scales did not properly assess the negative symptoms, especially the motivation-related ones; 2) the new scales which were developed to better assess negative symptoms were only available in research settings; 3) experienced psychiatrists involved ECPs’ training were not aware of the new negative symptoms definition and assessment methods.

Finally, the vast majority (almost 90%) of the studied sample agreed that there is a need for more training on the negative symptoms of SZ in the specialist training curriculum. This bottom-up expression of ECPs’ need of improved education is in line with the recent call to action for SZ care pathways, published by the European Brain Council, especially for enhancement of the workforce capacity and training (31), and needs to be addressed by appropriate authorities.

### 4.1 Limitations

The crucial limitation of the study protocol is the fact that the obtained results will be based on small, non-randomized, convenience sample of ECPs. Unfortunately, that is the limitation of many previous studies using a similar methodology (international European survey) for several reasons such as 1) the fact that the number of trainees in psychiatry/psychiatrists (per 10–000 citizens) varies greatly among European countries from 1.4 in Romania or Hungary to 5.3 in Switzerland or Nordic countries (32); 2) lack of representation for some European countries among EPA and EFPT structures and/or lack of response of NPA representatives upon invitation to collaborate on the CARE project; 3) difficulty engaging ECPs in study completion especially in countries with limited number of psychiatrists, in which they are likely confronting higher workloads or in countries in which the ECPs are not widely involved with NPAs. Nonetheless, the thresholds for sample sizes implemented in the CARE study are higher than the thresholds noted for previous international online surveys (33) and the sample sizes noted in already completed studies of similar design (34). Moreover, the inclusion of non-randomized sample might result in a selection bias, given that the ECPs might be more likely to respond to a survey which focuses on an issue toward which they are already sensitized. To limit this bias, the survey included questions allowing to control for training characteristics, which could affect the levels of knowledge and self-reported competence in negative symptoms evaluation and management.

This pilot analysis is based on a limited, non-randomized, convenience sample of ECPs; therefore, the obtained results might not be representative of the general ECP sample in Poland. Nonetheless, the pilot sample is relatively large compared to many other studies which followed a similar methodology (33–36).

## 5 Conclusions

The CARE study was designed to explore the competence and confidence of ECPs from many European countries in the evaluation and management of the negative symptoms of SZ. This might help informing future actions by the local and national institutions responsible for specialist training in psychiatry in Europe, by professional organizations striving to enhance the professional development of psychiatrists, as well as other entities aiming to improve the outcomes of people with SZ. These pilot results from the Polish sample reveal substantial deficits in knowledge and practical skills related to negative symptoms, alongside a strong demand for improved training. The forthcoming analysis of the full study results will provide data on these issues in the general European ECP sample, in order to gain a deep understanding of future educational needs for the optimization of SZ care in the domain of negative symptoms.

## Data Availability

The raw data supporting the conclusions of this article will be made available by the authors, without undue reservation.
