# Supplementary material for: The CARE project – study protocol and pilot results from the Polish population
Source: Front Psychiatry. 2025 Jul 23;16:1643722. doi: 10.3389/fpsyt.2025.1643722 (PMC12325343; doi:10.3389/fpsyt.2025.1643722)
Supplement: Supplementary file 1 [file Table1.docx]

Supplementary data. The CARE study survey

1. What is your gender?
2. Male
3. Female
4. Non-binary
5. Prefer not to disclose
6. What is Your professional status:
7. Trainee in general adult psychiatry
8. Trainee in child and adolescent psychiatry
9. Specialist in general adult psychiatry with less than 5 years of clinical practice after specialty
10. Specialist in child and adolescent psychiatry with less than 5 years of clinical practice after specialty
11. Specialist in general adult psychiatry under 40 years of age
12. Specialist in child and adolescent psychiatry under 40 years of age
13. What country do you work in?:

- Participant selects a response from a list of all countries of the WHO European region countries

1. What is your work experience in mental health care system (in years)?

- Participant selects a response from a list of 1-15 or more

1. Are You engaged in clinical research (i.e. PhD students, clinical investigators, co-authors of scientific publications)
2. Yes
3. No
4. Has the curriculum of your specialist training included theoretical courses on the negative symptoms of schizophrenia?
5. Yes
6. No
7. Has the curriculum of your specialist training included placements in specialist schizophrenia clinics / wards?
8. Yes
9. No
10. Have you participated in additional theoretical or practical training in the negative symptoms of schizophrenia assessment and management (outside of your specialist training programme) i.e. conference lectures or workshops, webinars, summer schools etc.?
11. Yes
12. No

9. Do You agree with the statement “I feel as competent in consulting people with negative

symptoms of schizophrenia, as I feel about consulting people with positive symptoms of

schizophrenia”?

1: Strongly disagree

2: Disagree

3: Neither disagree nor agree

4: Agree

5: Strongly agree

(assessed with a Likert-type scale ranging from 1 Strongly disagree to 5 Strongly agree).

10. Please indicate how competent You feel about consulting people with different psychiatric

diagnoses:

a) Major depressive disorder

b) Schizophrenia

c) Bipolar disorder

d) Personality disorder

e) Schizophrenia with persistent negative symptoms

f) Anxiety disorder

g) Substance dependence

1: Very incompetent

2: Incompetent

3: Neither competent nor incompetent

4: Competent

5: Very competent

(assessed with a Likert-type scale ranging from 1 Very incompetent to 5 Very competent).

11. Please indicate how willing would You be to provide a consultation to people with different

psychiatric diagnoses:

a) Major depressive disorder

b) Schizophrenia

c) Bipolar disorder

d) Personality disorder

e) Schizophrenia with persistent negative symptoms

f) Anxiety disorder

g) Substance dependence

1: Very unwilling

2: Unwilling

3: Neither willing nor unwilling

4: Willing

5: Very willing

(assessed with a Likert-type scale ranging from 1 Very unwilling to 5 Very willing).

12. Which symptoms are included in the main negative symptom domains according to the National

Institute of Mental Health–Measurement and Treatment Research to Improve Cognition in

Schizophrenia (NIMH–MATRICS) consensus statement and 2021 European Psychiatric

Association guidance on the assessment and treatment of negative symptoms? Choose as many

answers as You believe are correct.

(multiple choice, allows for selection of as many answers as the participant desires)

- - inappropriate affect
  - anhedonia
  - Asociality
  - difficulty in abstract thinking
  - anergy
  - alogia
  - mannerism and posturing
  - poverty of speech content
  - blunted affect
  - attentional impairment
  - avolition
  - disorganization
  - stereotyped thinking

13. Which tools for the assessment of the negative symptoms of schizophrenia do You feel well-

trained to administer and interpret? Choose as many answers as You believe are correct.

(multiple choice, allows for selection of as many answers as the participant desires)

a) Positive and Negative Syndrome Scale

b) Brief Psychiatric Rating Scale

c) Scale for the Assessment of Negative Symptoms

d) Brief Negative Symptom Scale

e) Clinical Assessment Interview for Negative Symptoms

f) Negative Symptom Assessment Scale

g) other

h) none

14. How often do You use tools mentioned in the above question during a clinical assessment to

monitor the course of negative symptoms of schizophrenia?

1: Almost never

2: Rarely

3: Sometimes

4: Often

5: Almost always

(assessed with a Likert-type scale ranging from 1 Almost never to 5 Almost always).

15. Which pharmacological intervention would You choose as first-line for a patient with

schizophrenia treated with haloperidol, who presents with negative symptoms, according to the

European Psychiatric Association guidance?

a) add-on antidepressant

b) combination with a dopamine partial-agonist antipsychotic

c) switch to a second-generation antipsychotic

16. Which non-pharmacological interventions would be recommended for a patient with

schizophrenia who presents with negative symptoms according to the most recent European

Psychiatric Association guidance? Choose as many answers as You believe are correct.

(multiple choice, allows for selection of as many answers as the participant desires)

a) social skills training

b) exercise

c) cognitive remediation

d) community interventions

e) supported employment and housing

17. What is the correct choice in case of moderate severity positive symptoms and concomitant

negative symptoms in subjects with more than one treatment failure in the current episode?

a) Increase the dose of the current antipsychotic

b) Switch to another antipsychotic of a different class

c) Add an antidepressant

d) Switch to clozapine

e) None of the above

18. Which nutraceuticals could be recommended as adjunctive treatment for a patient with

schizophrenia who presents with negative symptoms according to the 2022 World Federation of

Societies of Biological Psychiatry (WFSBP) and Canadian Network for Mood and Anxiety

Treatments (CANMAT) Taskforce Clinician guidelines for the treatment of psychiatric disorders

with nutraceuticals and phytoceuticals?

a) omega-3 fatty acids

b) methylfolate

c) both

d) none

19. Would You agree with the statement “I feel competent in evaluating the negative symptoms of

schizophrenia”?

1: Strongly disagree

2: Disagree

3: Neither disagree nor agree

4: Agree

5: Strongly agree

(assessed with a Likert-type scale ranging from 1 Strongly disagree to 5 Strongly agree).

20. Would You agree with the statement “I feel competent in managing the negative symptoms of

schizophrenia?

1: Strongly disagree

2: Disagree

3: Neither disagree nor agree

4: Agree

5: Strongly agree

(assessed with a Likert-type scale ranging from 1 Strongly disagree to 5 Strongly agree).

21. Are You familiar with the 2021 European Psychiatric Association (EPA) guidance on the

assessment and treatment of negative symptoms?

a) yes

b) no

22. Are You familiar with the 2022 World Federation of Societies of Biological Psychiatry (WFSBP)

and Canadian Network for Mood and Anxiety Treatments (CANMAT) Taskforce Clinician

guidelines for the treatment of psychiatric disorders with nutraceuticals and phytoceuticals?

a) yes

b) no

23. Would You agree with the statement “specialist training should put more emphasis and/or time on

the evaluation and management of the negative symptoms of schizophrenia”?

1: Strongly disagree

2: Disagree

3: Neither disagree nor agree

4: Agree

5: Strongly agree

(assessed with a Likert-type scale ranging from 1 Strongly disagree to 5 Strongly agree).

Thank You for Your answers! Now, You can find the correct responses and source publications below.

Response to question 11: Which symptoms are included in the main negative symptom domains according to the National Institute of Mental Health–Measurement and Treatment Research to Improve Cognition in Schizophrenia (NIMH–MATRICS) consensus statement and 2016 European Psychiatric Association guidance on the assessment and treatment of negative symptoms?

“As currently understood, the domains of negative symptoms include blunted affect, alogia, asociality, anhedonia, and avolition. “ ^1,2^

Response to question 12: Which tools for the assessment of the negative symptoms schizophrenia do You feel well-trained to administer?

The guidance on the evaluation of negative symptoms of schizophrenia is depicted in the Figure from EPA guidance on assessment of negative symptoms in schizophrenia ^3^

Response to question 15: Which pharmacological intervention would You choose as first-line for a patient with schizophrenia treated with haloperidol, who presents with negative symptoms? The correct answer is “c: switch to a second-generation antipsychotic”. According to the EPA guidance on the treatment of negative symptoms in schizophrenia^4^:

“For patients with negative symptoms who are treated with a first- generation antipsychotic, a switch to a second-generation antipsychotic should be considered” ^4^

“If negative symptoms do not improve after optimization of antipsychotic treatment, a trial with an add-on antidepressant should be considered for patients with negative symptoms after careful evaluation of risks and benefits” ^4^

“Guidelines recommend antipsychotic monotherapy for the treatment of schizophrenia” ^4^

Response to question 16 “Which non-pharmacological interventions would be recommended for a

patient with schizophrenia who presents with negative symptoms according to the 2021 European Psychiatric Association? All answers as are correct.

“Social skills training should be offered to patients with negative symptoms, but no specific

recommendation for patients with primary negative symptoms can be given. Furthermore, the

available evidence does not allow recommending one specific program for social skills training.” ^4^

“Cognitive remediation can be considered for patients with negative symptoms, in particular

for those who also show cognitive impairment.” ^4^

“Exercise can be considered for persons suffering from negative symptoms as part of an

integrated treatment plan also aiming at improving physical health.” ^4^

“The access to care for patients with negative symptoms should have a low-threshold and

should be facilitated by assertive community interventions.” ^4^

“Patients with negative symptoms should have access to rehabilitation interventions such as

supported employment and supported housing.” ^4^

Response to question 17 “What is the correct choice in case of residual positive symptoms and concomitant negative symptoms in subjects with more than one treatment failure in the current episode”? The correct answer is „d Switch to clozapine,” as more than one treatment failures for positive symptoms are observed, and subjects present with moderate, severe positive symptoms, which require, according to present guidelines, the switch to clozapine. According to the EPA guidance on the treatment of negative symptoms in schizophrenia^4^:

“Although recent meta-analyses showed somewhat inconsistent results, clozapine remains the

main recommendation for treatment-resistant positive symptoms” ^4^

“In a patient presenting with negative symptoms that are considered to be secondary to

treatment-resistant positive symptoms, a trial with clozapine should be considered” ^4^

Response to question 18 “Which nutraceuticals would be recommended for a patient with schizophrenia who presents with negative symptoms? The correct answer is “c: both”, according to the World Federation of Societies of Biological Psychiatry (WFSBP) and Canadian Network for Mood and Anxiety Treatments (CANMAT) Taskforce ^5^:

“Omega-3 fatty acids at doses of 1 g to 2 g are Not Recommended for Adjunctive or Monotherapy use in schizophrenia” ^5^

“Methylfolate (1 mg to 15 mg) per day is Provisionally Recommended for Adjunctive use in schizophrenia (primarily for negative symptoms)” ^5^

1. Kirkpatrick B, Fenton WS, Carpenter WT, Marder SR. The NIMH-MATRICS consensus statement on negative symptoms. *Schizophrenia Bulletin*. 2006;32(2):214-219. doi:10.1093/schbul/sbj053

2. Galderisi S, Mucci A, Buchanan RW, Arango C. Negative symptoms of schizophrenia: new developments and unanswered research questions. *The Lancet Psychiatry*. 2018;5(8):664-677. doi:10.1016/S2215-0366(18)30050-6

3. Galderisi S, Mucci A, Dollfus S, et al. EPA guidance on assessment of negative symptoms in schizophrenia. *European Psychiatry*. 2021;64(1). doi:10.1192/j.eurpsy.2021.11

4. Galderisi S, Kaiser S, Bitter I, et al. EPA guidance on treatment of negative symptoms in schizophrenia. *European Psychiatry*. 2021;64(1):e21. doi:10.1192/j.eurpsy.2021.13

5. Sarris J, Ravindran A, Yatham LN, et al. Clinician guidelines for the treatment of psychiatric disorders with nutraceuticals and phytoceuticals: The World Federation of Societies of Biological Psychiatry (WFSBP) and Canadian Network for Mood and Anxiety Treatments (CANMAT) Taskforce. *World Journal of Biological Psychiatry*. 2022;23(6):424-455. doi:10.1080/15622975.2021.2013041
